# Diversity investigation by application of DNA barcoding: A case study of lepidopteran insects in Xinjiang wild fruit forests, China

**DOI:** 10.1002/ece3.8678

**Published:** 2022-03-07

**Authors:** Jinyu Zhan, Yufeng Zheng, Qing Xia, Jin Wang, Sibo Liu, Zhaofu Yang

**Affiliations:** ^1^ 12469 Key Laboratory of Plant Protection Resources and Pest Management Ministry of Education Northwest A&F University Yangling China; ^2^ 12469 Entomological Museum College of Plant Protection Northwest A&F University Yangling China

**Keywords:** DNA barcoding, lepidopteran insects, species diversity, species identification, Xinjiang wild fruit forests

## Abstract

To investigate the species diversity of lepidopteran insects in Xinjiang wild fruit forests, establish insect community monitoring systems, and determine the local species pool, we test the applicability of DNA barcoding based on cytochrome *c* oxidase subunit I (*COI*) gene for accurate and rapid identification of insect species. From 2017 to 2019, a total of 212 samples with ambiguous morphological identification were selected for DNA barcoding analysis. Five sequence‐based methods for species delimitation (ABGD, BINs, GMYC, jMOTU, and bPTP) were conducted for comparison to traditional morphology‐based identification. In total, 2,422 samples were recorded, representing 143 species of 110 genera in 17 families in Lepidoptera. The diversity analysis showed that the richness indices for Noctuidae was the highest (54 species), and for Pterophoridae, Cossidae, Limacodidae, Lasiocampidae, Pieridae, and Lycaenidae were the lowest (all with 1 species). The Shannon–Wiener species diversity index (H′) and Pielou's evenness (J′) of lepidopteran insects first increased and then decreased across these 3 years, while the Simpson diversity index showed a trend of subtracted then added. For molecular‐based identification, 67 lepidopteran species within 61 genera in 14 families were identified through DNA barcoding. Neighbor‐joining (NJ) analysis showed that conspecific individuals were clustered together and formed monophyletic groups with a high support value, except for *Lacanobia contigua* (Denis & Schiffermüller, 1775) (Noctuidae: Hadeninae). Sixty‐seven morphospecies were classified into various numbers of MOTUs based on ABGD, BINs, GMYC, jMOTU, and bPTP (70, 96, 2, 71, and 71, respectively). In Xinjiang wild fruit forests, the family with the largest number of species is Noctuidae, followed by Geometridae, Crambidae, and the remaining families. The highest Shannon diversity index is observed for the family Noctuidae. Our results indicate that the distance‐based methods (ABGD and jMOTU) and character‐based method (bPTP) outperform GMYC. BINs is inclined to overestimate species diversity compared to other methods.

## Graphical abstract

We investigate the species diversity of lepidopteran insects in Xinjiang wild fruit forests to establish insect community monitoring systems as well as determine the local species pool, by testing the applicability of DNA barcoding based on mtDNA COI gene for accurate and rapid identification of insect species.

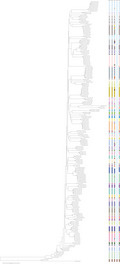

## 1 INTRODUCTION

Xinjiang wild fruit forests is located in the eastern end of the Tianshan belt in central Asia, and is distributed mainly in the Yili River Valley region (Lin et al., 2006). It is a special broad‐leaved forest comprised of a mixture of paleotemperate broad‐leaved forests and northern forest meadows, and is a priority conservation ecosystem in China (Cheng et al., 2020; Xu et al., 2006). This region has high species diversity and a relatively complex community structure, and is an important ingredient of Xinjiang's biodiversity (Li et al., 2011). Due to the uniqueness of the community, the Xinjiang wild fruit forests have been included in the list of China's priority ecosystems (Yang et al., 2003), containing a natural gene pool which has extremely high genetic and species diversity. However, due to the unique local climate, geographical conditions, and characteristics of vegetation evolution, coupled with human factors including agricultural reclamation, tourism development, overgrazing, etc., this area of Xinjiang wild fruit forests has decreased sharply and this ecosystem has been seriously damaged. In particular, the self‐regulation and recovery abilities of the Xinjiang wild fruit forest ecosystem are delicate (Ding, 2007; Fang et al., 2019).

Insects are an important constituent of biodiversity and play a very significant role in maintaining the structure and function of forest ecosystems (Springett, 1978). Lepidoptera is the second largest order of Insecta, which makes it an extremely important indicator in monitoring and assessing biodiversity. About 130,000 to 160,000 species of butterflies and moths are known in the world, occupying about 15% of insect diversity (Blair & Launer, 1997; McKinney, 2008). In recent years, wild fruit forests have been degenerating due to major threats outside of human disturbance. For example, the larvae of *Agrilus mali* Matsumura (Coleoptera: Buprestidae) is inflicting serious damage to the trunk cortex of *Malus sieversii* (Ledeb.) M. Roem. (Rosales: Rosaceae), which is an endangered and key protected apple tree species in China (Cui et al., 2015; Wang, Zhang, Yang, et al., 2013; Zhang et al., 2018). Additionally, other lepidopteran pests that also extensively feed on various plants and crops in Xinjiang wild fruit forests cause serious damage to the wild fruit forest ecosystem (Hei et al., 2016; Zhang et al., 2019; Zhou, Dong, et al., 2020). Therefore, the study of the diversity of lepidopteran insects in Xinjiang wild fruit forests plays an important role in monitoring the changes in wild fruit forest ecosystem. At the same time, it is vital to explore the effects of environmental factors (e.g., temperature and precipitation) and external disturbance factors (e.g. pests, tourism, and animal husbandry) on the biodiversity of the Xinjiang wild fruit forests.

Traditional morphological classification methods have been used to describe the diversity of life on Earth (Packer et al., 2009). However, this traditional method requires a great deal of expertise and is laborious. In addition, it may be difficult to identify damaged specimens or distinguish closely related species that are morphologically similar. DNA barcoding is a complementary identification method to morphological identification, and assists in distinguishing morphological similar species (Batovska et al., 2016). In other words, DNA barcoding has the distinct advantage of being independent of morphological characteristics by using one or several short‐standardized DNA regions for species identification (Hebert, Cywinska, et al., 2003; Kress et al., 2005; Ren et al., 2019; Yang et al., 2018). It has become an important scientific approach for understanding world biodiversity (Elías‐Gutiérrez & León‐Regagnon, 2013). Some early critics have proposed limits to DNA barcoding, attributed to the variation in standard threshold for species discrimination in some groups, the presence of nuclear mitochondrial pseudogenes (Numts), and incomplete lineage sorting (Hickerson et al., 2006; Meyer & Paulay, 2005; Wheeler, 2005). Others view this approach as an additional and useful tool for species identification (Schindel & Miller, 2005; Tautz et al., 2003) and an effective method for a variety of applications that involve the identification of species (Boissin et al., 2017; Bozorov et al., 2019; Liu et al., 2014; Yang, Zhai, et al., 2016; Yang, Landry, et al., 2016).

In this study, we employ both the traditional morphological method and DNA barcoding technology through analysis of the mitochondrial marker cytochrome *c* oxidase subunit I (*COI*) gene to determine species diversity of the Xinjiang wild fruit forests over 3 consecutive years. The species diversity was analyzed and the influence of external factors on the wild fruit forest ecosystem was explored in order to illuminate the composition and dynamic changes of the insect community in this special ecosystem. Our study not only establishes the preliminarily insect monitoring system and local species gene pool of the Xinjiang wild fruit forests but also provides a scientific basis for ecological recovery and conservation measures of the wild fruit forests in the future, as well as a basis for rational development and utilization of wild fruit forest resources today.

## 2 MATERIALS AND METHODS

### 2.1 Study area

The study site is located at the southern edge of the Tianshan Mountains. Samples were collected in a chessboard‐type pattern on the north slope of the wild fruit forest of Xinyuan County in Xinjiang. A 100 m × 100 m area between 1,380 m and 1,450 m altitude was divided into 25 small sample plots of 20 m × 20 m. The latitude and longitude range of the wild fruit forests are 80°42′52″–83°37′17″E and 43°13′14″–44°26′28″N, respectively, and the altitudes of these sites are 1,100–1,500 m (Cheng et al., 2019; Zhou, Zhao, et al., 2020). This region has an annual average sunshine duration of 2898.4 h and an annual average temperature of 10.4°C (Kong et al., 2017; Wang, Zhang, Wei, et al., 2013). The annual average precipitation ranges within 260–800 mm, making it the wettest region in Xinjiang (Tian et al., 2012).

### 2.2 Sample collection and identification

Samples were collected by light trapping and net catching in 25 sample plots during July from 2017 to 2019. In order to avoid the interference of subjective factors, two 20W ultraviolet lamps were used to collect samples from 22:00 to 02:00 at fixed sample collection sites in every collecting year. During the day, samples were collected in the checkerboard pattern using sweeping nets in a 100 × 100 m area. The specimens were sorted, collected information was recorded, and partial samples of each species were placed in triangular collection papers. These samples were carried back to the laboratory where pinned dry specimens were used to carry out morphological identification using available taxonomic references. Remaining specimens were preserved in 100% ethanol and stored at −20°C for molecular identification based on the mitochondrial *COI* gene. In this study, 212 samples with ambiguous morphological identification were selected for DNA barcoding analysis. All specimens were deposited in the Entomological Museum, Northwest A&F University (NWAFU), Yangling, Shaanxi, China.

### 2.3 Diversity analysis

Data were analyzed following Subedi et al. (2021). We calculated the Shannon–Weiner diversity index (H′) (Shannon & Weaver, 1949), Simpson index (S′) (Simpson, 1949), species richness, and Pielou's evenness (J′) (Pielou, 1966). Simpson index ranges from 0 to 1 with 0 representing infinite diversity and 1 representing no diversity. Species richness was used for a count of species observed in a sample area. Pielou's evenness index was used for determining the evenness of species in the community (Subedi et al., 2021).

Species richness was defined as the number of species. Simpson index (S′), Shannon–Wiener index (H′), and Pielou's evenness index (J′) were calculated with the following equations: S′ = ∑*N_i_* (*N_i_* − 1)/*N* (*N* − 1), H′ = −∑*P_i_* In*P_i_* (*P_i_* = *N_i_*/*N*), J′ = H′/ln*S*, respectively. *S* is the number of species, *N_i_* is the number of individuals of the *i* ^th^ species, *N* is the total number of individuals of all species, and *P_i_* is the proportion of individuals of the *i* ^th^ species in the community.

### 2.4 DNA extraction, PCR, and sequencing

Genomic DNA extraction was carried out using one or two legs of adult specimens by the DNeasy DNA Extraction kit (TransGen Biotech, Beijing, China), following the manufacturer's protocol. The fragment of mitochondrial *COI* gene was amplified using the primers LCO1490 (5′‐GGCTCAACAAATCATAAAGATATTGG‐3′) and HCO2198 (5′‐TAAACTTCAGGGTGACCAAAAAATCA‐3′) (Folmer et al., 1994). PCR reactions were performed in a total volume of 25 μl using 2 μl of DNA extract, 1 µl each of forward and reverse primer, 12.5 μl Green‐Mix, and 8.5 μl ddH_2_O. The reaction cycle consisted of an initial 1 min at 94°C, followed by a pre‐amplification step of 5 cycles of 94°C for 1 min, 94°C for 1.5 min, 45°C for 1.5 min, 72°C for 1 min, an amplification step of 30 cycles of 94°C for 1.5 min, 51°C for 1.5 min, and 72°C for 1 min with a final extension of 72°C for 5 min. PCR products were separated by electrophoresis in a 1% agarose gel, and sequencing was performed at AuGCT Biotech (Beijing, China) using the same primers as in the PCR.

### 2.5 Sequence analysis

Sequences generated in this study were assigned through a similarity search against the GenBank public database (https://www.ncbi.nlm.nih.gov/) and BOLD system (Barcode of Life Data System) (http://www.boldsystems.org/). A reference sequence library was then constructed for each species with sequences with 98%–100% similarity (Hosein et al., 2017; Larranaga & Hormaza, 2015). Contaminated sequences were excluded in this study. Multiple sequence alignments were generated using Clustal X 2.0 (Larkin et al., 2007). MEGA v10.0 software was used to calculate intraspecific and interspecific genetic divergences (Kumar et al., 2018). Two sequences from the order Trichoptera were downloaded from GenBank (GU114778.1 & GU711425.1) and were used as outgroups. Neighbor‐joining (NJ) analyses was carried out using the Kimura 2‐parameter (K2P) molecular evolutionary model with 1000 bootstrap replications in MEGA v10.0 (Hebert, Cywinska, et al., 2003; Hebert, Ratnasingham, et al., 2003; Kumar et al., 2018; Liu et al., 2014; Saitou & Nei, 1987). The K2P was chosen because it takes into account the multiple effects of transformation and transversion, and provides base substitution patterns that are very similar to mitochondrial DNA, allowing relatively accurate interspecific and intraspecific genetic distances to be obtained (Čandek & Kuntner, 2015; Nei & Kumar, 2000).

### 2.6 Species delimitation analysis

In the present study, we compared comprehensively the performance of five species delimitation methods for Molecular Operational Taxonomic Units (MOTUs) designations, Automatic Barcode Gap Discovery (ABGD) (Puillandre et al., 2012), Barcode Index Number system (BINs) (Ratnasingham & Hebert, 2013; Yang, Landry, et al., 2016), a Java program that uses an explicit, determinate algorithm to define Molecular Operational Taxonomic Unit (jMOTU) (Jones et al., 2011; Zhou et al., 2019), General Mixed Yule Coalescent (GMYC) (Fujisawa & Barraclough, 2013; Pons et al., 2006), and Bayesian Poisson Tree Processes (bPTP) (Zhang et al., 2013) to examine the correspondence between morpho‐based and molecular‐based species identifications.

We performed the ABGD analysis at the available website (https://bioinfo.mnhn.fr/abi/public/abgd/abgdweb.html). The following parameters were used to run the program: Pmin = 0.001, Pmax = 0.1, Steps = 10, X (relative gap width) = 1.0, and Nb bins (for distance distribution) = 20. Three different distance models, the Jukes‐Cantor, Kimura 2‐parameter (K2P), and Simple Distance (p‐distance), were conducted (Puillandre et al., 2012). BINs were generated automatically in the BOLD system (http://www.boldsystems.org/), which is based on a distance method to cluster DNA barcodes to generate OTUs. We used jMOTU, which is a distanced‐based method, to generate MOTUs through a Java program (Jones et al., 2011). The analysis was implemented in jMOTU1.0.7 with a cut‐off value of 95% (Gao et al., 2021). We employed the GMYC and bPTP, which are both based on phylogenetic trees, to classify species into MOTUs. For GMYC analyses, an ultrametric gene tree obtained through BEAST v1.8.0 was uploaded to GMYC web server (https://species.h‐its.org/gmyc/) with two models (single threshold and multiple thresholds, respectively) (Fujisawa & Barraclough, 2013; Pons et al., 2006; Sari et al., 2016). For bPTP analyses, a maximum likelihood tree was input in bPTP web server (https://species.h‐its.org/ptp/) and MOTUs were generated in highest Bayesian solution and maximum likelihood solution with the default parameters (no. MCMC generations = 100,000, thinning = 100, burn‐in = 0.1, and seed = 123) (Saddhe et al., 2017; Zhang et al., 2013).

## 3 RESULTS

### 3.1 Composition characteristics of lepidopteran insects

**FIGURE 1:**
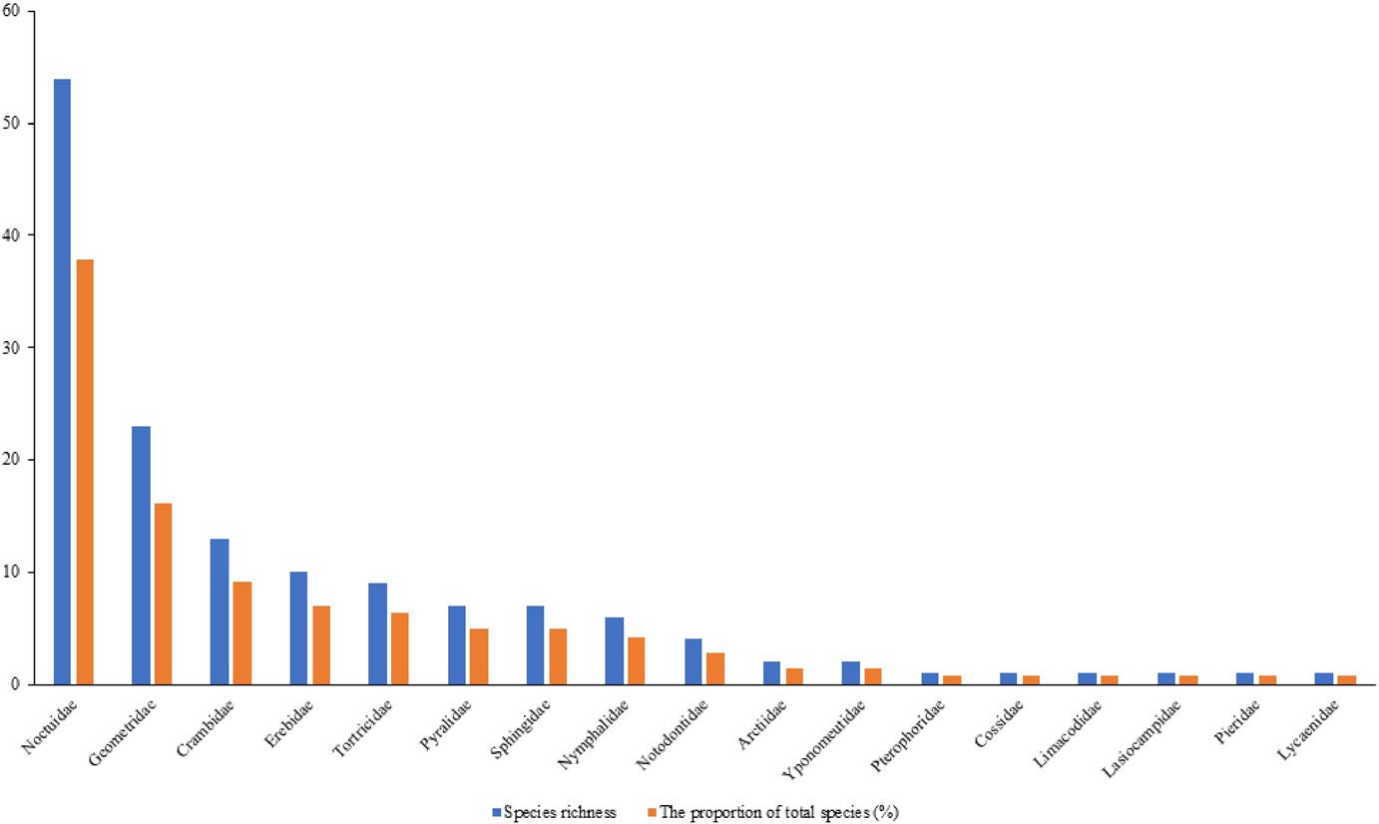
Analysis of diversity, species richness, and the proportion of total species of lepidopteran insects in Xinjiang wild fruit forests (2017–2019)

In total, 2,422 individuals were sampled, representing 143 species determined by morphological identification belonging to 110 genera in 17 families in Lepidoptera (Table 1). The family with the most observed individuals was Noctuidae (1,126 individuals), followed by Pyralidae (263 individuals) and Crambidae (259 individuals). Cossidae and Lycaenidae had the least number (only one individual each). The numbers of individuals observed in the remaining 12 families, in order from most numerous, were in Erebidae, Notodontidae, Geometridae, Sphingidae, Tortricidae, Pieridae, Nymphalidae, Pterophoridae, Arctiidae, Yponomeutidae, Lasiocampidae, and Limacodidae. The species richness index was used to determine lepidopteran insects’ diversity. Our results show that the species richness index of Noctuidae is the highest (54 species), where the proportion of total species is 37.76%. Two families, Geometridae (23 species) and Crambidae (13 species), were observed at a relatively high ratio (16.08% and 9.09%, respectively). By contrast, Pterophoridae, Cossidae, Limacodidae, Lasiocampidae, Pieridae, and Lycaenidae were the lowest (all with 1 species, 0.70%). The proportion of species observed in the remaining eight families are in the order: Erebidae (10 species, 6.99%), Tortricidae (9 species, 6.29%), Pyralidae (7 species, 4.90%), Sphingidae (7 species, 4.90%), Nymphalidae (6 species, 4.20%), Notodontidae (4 species, 2.80%), Arctiidae (2 species, 1.40%), and Yponomeutidae (2 species, 1.40%) (Figure 1).

**TABLE 1:** Composition characteristics of each family of lepidopteran insects observed in Xinjiang wild fruit forests (2017–2019)

Taxa	Number of genera	Number of species	Number of individuals
Noctuidae	38	54	1126
Geometridae	19	23	152
Crambidae	9	13	259
Tortricidae	9	9	32
Erebidae	8	10	257
Pyralidae	6	7	263
Sphingidae	5	7	94
Notodontidae	3	4	187
Arctiidae	1	2	4
Yponomeutidae	1	2	4
Pterophoridae	1	1	5
Cossidae	1	1	1
Limacodidae	1	1	3
Lasiocampidae	1	1	4
Nymphalidae	5	6	13
Pieridae	1	1	17
Lycaenidae	1	1	1
Total	110	143	2422

### 3.2 Species diversity and abundance of lepidopteran insects

**FIGURE 2:**
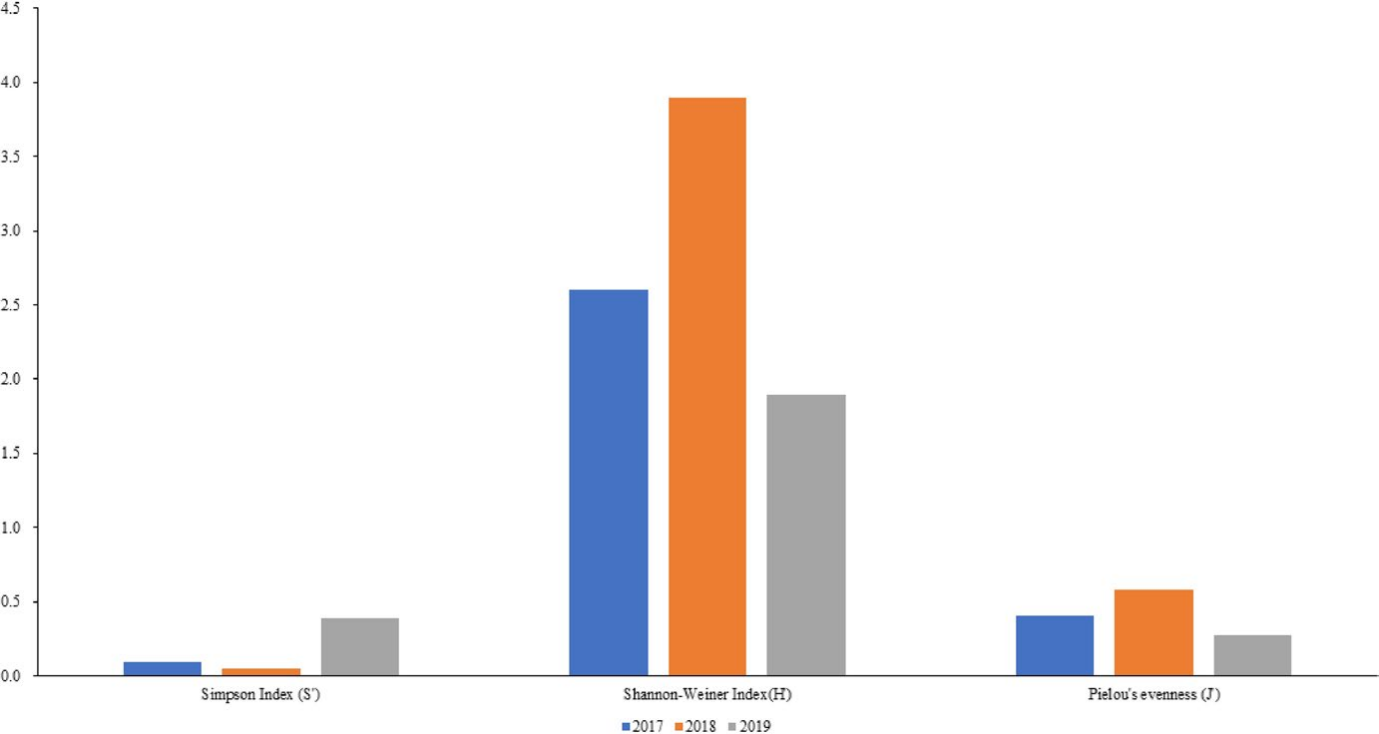
Analysis of diversity indices (Shannon–Weiner diversity index, Simpson diversity index, and Pielou's evenness) showing trends of lepidopteran insects in Xinjiang wild fruit forests (2017–2019)

The results of the 3 years of analysis of diversity (Table 2) show that the Simpson index (S′), Shannon–Wiener index (H′), and Pielou's evenness index (J′) of lepidopteran insects in the Xinjiang wild fruit forests in 2017 were 0.0948, 2.6033, and 0.4058, respectively. In 2018, Simpson index (S′), Shannon–Wiener index (H′), and Pielou's evenness index (J′) of lepidopteran insects were 0.0451, 3.8982, and 0.5829, respectively. In 2019, these three indices were 0.3903, 1.8938, and 0.2738, respectively. Diversity indices for each family in the Xinjiang wild fruit forests are summarized in Table 2. According to species diversity and abundance analysis of 3 years of lepidopteran insects in the Xinjiang wild fruit forests (Figure 2), the Shannon–Wiener index and Pielou's evenness index both showed a trend of added then subtracted, while the Simpson diversity index showed a trend of subtracted then added.

**TABLE 2:** Descriptive measures of analysis (Shannon–Weiner diversity index, Simpson diversity index, species richness, and Pielou evenness) calculated for each family of lepidopteran insects observed in Xinjiang wild fruit forests, as well as the overall values when data from all families were pooled together (2017–2019)

Family	Year	Individuals	Species	Simpson Index (S′)	Shannon–Wiener Index (H′)	Pielou's evenness (J′)
Noctuidae	2019	763	25	0.6712	0.9787	0.3041
	2018	296	42	0.2075	2.5198	0.6742
	2017	67	5	0.4378	0.9370	0.5822
Geometridae	2019	47	4	0.3645	0.2419	0.1745
	2018	101	22	0.1170	2.5040	0.8101
	2017	4	2	0.3333	0.6931	0.5000
Crambidae	2019	60	6	0.4282	0.2772	0.1999
	2018	70	11	0.1470	2.0974	0.8747
	2017	129	4	0.3173	1.2321	0.8887
Tortricidae	2019	0	0			
	2018	32	9	0.0948	2.1424	0.9750
	2017	0	0			
Erebidae	2019	22	4	0.3766	0.1347	0.0972
	2018	149	10	0.2496	1.6243	0.7054
	2017	86	4	0.5587	0.7628	0.5503
Pyralidae	2019	52	2	0.5513	0.2261	0.3262
	2018	92	6	0.3712	1.2228	0.6825
	2017	119	3	0.4394	0.9167	0.8344
Sphingidae	2019	55	2	0.9286	0.2008	0.2897
	2018	21	6	0.4429	1.1531	0.946
	2017	18	2	0.8889	0.2146	0.3095
Notodontidae	2019	4	2	0.5000	0.0305	0.0440
	2018	8	2	0.5714	0.5623	0.8113
	2017	175	3	0.5766	0.6367	0.5795
Arctiidae	2019	0	0			
	2018	4	2	0.5000	0.5623	0.8113
	2017	0	0			
Yponomeutidae	2019	0	0			
	2018	4	2	0.3333	0.6931	1.0000
	2017	0	0			
Pterophoridae	2019	0	0			
	2018	0	0			
	2017	5	1	1.0000	0.0000	0.0000
Cossidae	2019	0	0			
	2018	1	1	0.0000	0.0000	0.0000
	2017	0	0			
Limacodidae	2019	0	0			
	2018	3	1	1.0000	0.0000	0.0000
	2017	0	0			
Lasiocampidae	2019	0	0			
	2018	4	1	1.0000	0.0000	0.0000
	2017	0	0			
Nymphalidae	2019	2	2	0.0000	0.0174	0.0251
	2018	11	6	0.1091	1.7202	0.9601
	2017	0	0			
Pieridae	2019	4	1	1.0000	0.0275	0.0000
	2018	6	1	1.0000	0.0000	0.0000
	2017	7	1	1.0000	0.0000	0.0000
Lycaenidae	2019	0	0			
	2018	0	0			
	2017	1	1	0.0000	0.0000	0.0000
Lepidoptera	2019	1009	46	0.3903	1.8968	0.2738
	2018	802	122	0.0451	3.8982	0.5829
	2017	611	26	0.0948	2.6033	0.4058

Shannon–Wiener index values of each family ranged from 0.0000 to 2.5198, among which the Noctuidae reached the highest value in 2018 (2.5198) (Table 2). Among 17 families in the Xinjiang wild fruit forests, the Shannon–Wiener index for Noctuidae, Geometridae, Crambidae, Erebidae, Pyralidae, and Sphingidae increased and then decreased, while that of Lycaenidae, Pterophoridae, Yponomeutidae, and Cossidae demonstrated no evident changes. By contrast, the Shannon–Wiener index of Notodontidae showed a decreasing trend while the index for Pieridae increased.

### 3.3 Sequenced species identifications

A total of 196 *COI* barcodes representing 67 species in 61 genera of 14 families were generated in this study (Appendix S1), including Noctuidae (23 species in 19 genera), Geometridae (8 species in 7 genera), Erebidae (7 species in 7 genera), Crambidae (7 species in 6 genera), Pyralidae (6 species in 6 genera), Tortricidae (5 species in 5 genera), Nymphalidae (3 species in 3 genera), Sphingidae (2 species in 2 genera), Lasiocampidae, Notodontidae, Yponomeutidae, Cossidae, Arctiidae, and Pieridae (1 species in 1 genus). All sequences were used in the subsequent analysis. The average length of obtained sequences was 677 bp. The average nucleotide compositions of the *COI* sequences were 29.75% T, 39.60% A, 15.34% C, and 15.31% G (Appendix S2). The content of A + T (69.35%) was higher than that of G + C (30.65%). The sequences were aligned with the corresponding reference species in the GenBank database (accession numbers in Appendix S1) with a similarity higher than 99.00%, and had been uploaded to GenBank (accessions: MZ686723–MZ686918). According to these molecular analyses, this DNA barcode database of lepidopteran insects in wild fruit forests of Xinjiang has been initially established.

### 3.4 Genetic distance analysis

The overall mean genetic distance was 15.70%, and pairwise genetic distances ranged from 0% to 35.15% (Appendix S3). Intraspecific mean sequence divergence ranged between 0% and 3.11% (0.57% on average), while interspecific divergence ranged from 3.52% to 33.65% (15.96% on average) (Appendix S4). The highest intraspecific mean genetic distance of 3.11% was found in *Mythimna farrago* (Fabricius, 1787) (Noctuidae: Hadeninae), followed by *Phragmatobia fuliginosa* (Linnaeus, 1758) (Erebidae: Arctiinae) (2.39%). The highest interspecific divergence of 33.65% was discovered between *Phycitodes binaevella* (Hübner, 1813) (Pyralidae: Phycitinae) and *Aglais urticae* (Linnaeus, 1758) (Nymphalidae: Nymphalinae).

### 3.5 Clustering analysis of NJ tree

**FIGURE 3:**
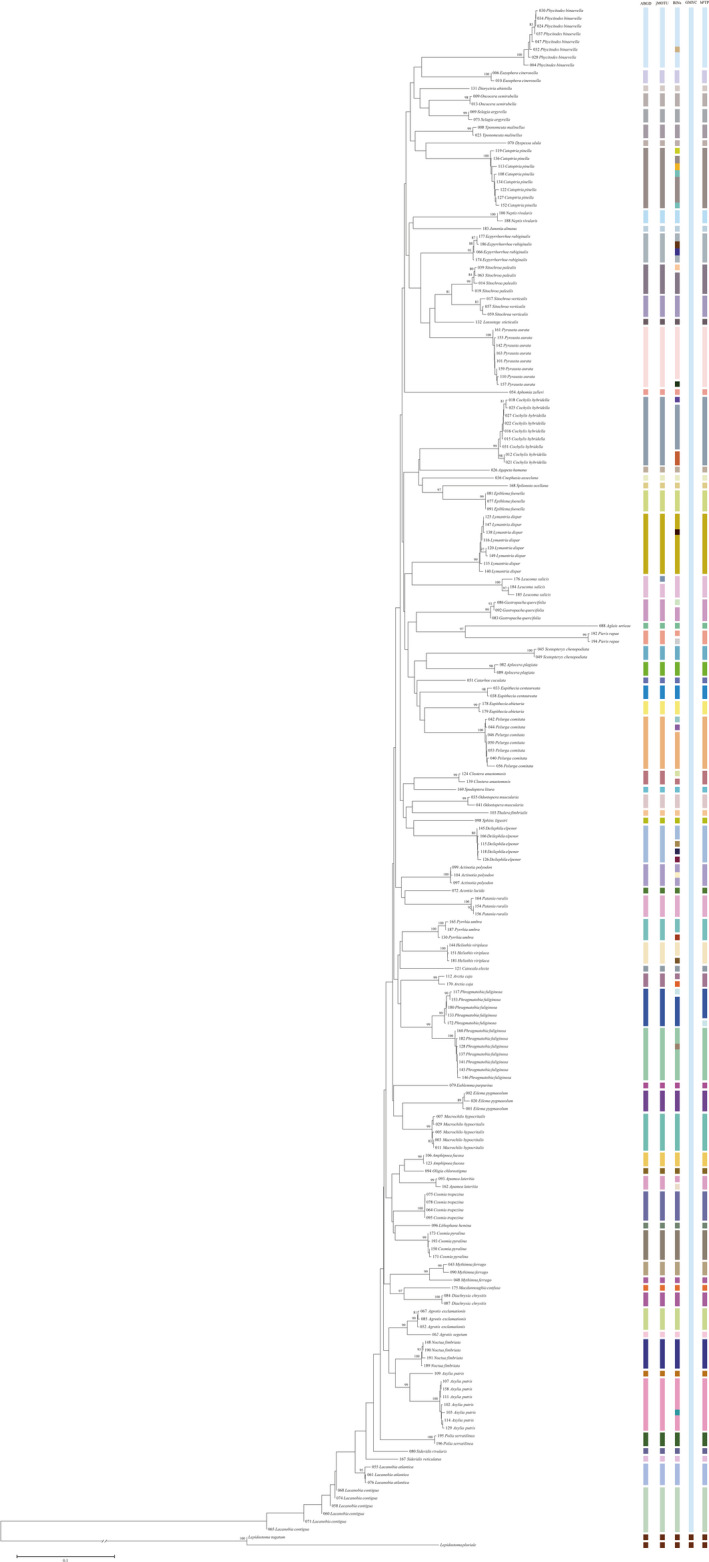
Neighbor‐joining (NJ) tree based on Kimura two‐parameter (K2P) model using 196 *COI* sequences of lepidopteran specimens sampled in this study and two trichopteran specimens downloaded from GenBank as outgroup. OTUs designations generated for 196 sequences of 67 morphospecies in five methods (ABGD, jMOTU, BINs, GMYC, and bPTP) in this study. Bootstrap is indicated above the branches. The right vertical bars show the results of species delimitation, with each MOTU represented by a different color block

The neighbor‐joining (NJ) analyses of the 196 *COI* sequence dataset (Appendix S2) revealed that conspecific individuals clustered together in most cases with high bootstrap support (more than 80%), except for *Lacanobia contigua* ([Denis & Schiffermüller], 1775) (Noctuidae: Hadeninae) (below 80%). And there was a clear separation among congeneric species (Figure 3). All species assignments are consistent with the morphological data. The DNA barcode database of Lepidoptera in wild fruit forests built in this study has a high efficiency in species identification and can be used for identifying lepidopterans accurately and rapidly in Xinjiang wild fruit forests.

### 3.6 Species identification and delimitation

Five methods examined in our study for species delimitations are shown in Figure 3. ABGD analysis results based on JC, K2P, and p‐distance showed significant DNA barcoding gaps (genetic distance thresholds were 0.01–0.03, 0.01–0.03, and 0.02–0.03, respectively), suggesting that the minimum interspecific genetic distance was greater than the maximum intraspecific genetic distance. The species partitioning results in the *P* interval of 0.001–0.1 prior values including both recursive partitioning and initial partitioning are shown in Table 3. The initial partitioning based on the three distance measures (JC, K2P, and p‐distance) all generated 70 MOTUs. Recursive partitioning based on p‐distance produced 70 MOTUs, while JC and K2P grouped in a range 70–86 MOTUs.

**TABLE 3:** Automatic partition produced by ABGD with three metrics (JC6_9_, K2P, and p‐distance)

Relative gap width	Prior maximal distance (*p*)	JC6_9_		K2P		p‐distance	
		Recursive partitions	Initial partitions	Recursive partitions	Initial partitions	Recursive partitions	Initial partitions
*X* = 1.0	.0359	0	0	1	1	0	0
	.0215	70	70	70	70	0	0
	.0129	70	70	70	70	0	0
	.0077	70	70	70	70	70	70
	.0046	70	70	70	70	70	70
	.0028	71	70	71	70	70	70
	.0017	71	70	71	70	70	70
	.0010	86	70	86	70	70	70

jMOTU analysis results showed that the number of MOTUs varied slightly within the sequence cut‐off values in 4–10 bp, and the range of MOTUs was 71–85. As the cut‐off value approached 95%, sequences were classified into 71 groups. These results show that the number of species was overestimated in comparison with morphological identification.

A total of 97 OTUs were obtained from 196 sequences of 67 morphospecies in the BOLD system using the Refined Single Linkage algorithm (RESL), of which 47 species were each assigned in a single BINs, 13 species were each assigned to two BINs, four species were each divided into three BINs, and three species were each separated into four BINs. In general, the number of OTUs was overestimated by BIN compared to morpho‐based identifications.

GMYC generated two ML clusters and two ML entities in the single‐threshold model, both with a confidence interval of 1–22. GMYC generated 44 ML clusters and 65 ML entities in the multiple threshold model, with confidence intervals of 19–48 and 23–91, respectively.

The bPTP analysis showed different results based on Bayesian and ML support. The ML method result corresponds to the PTP analysis result, and the Bayesian method corresponds to the bPTP analysis result. ML and BS methods recognized 71 MOTUs and 75 MOTUs, excluding the outgroups, respectively. The result of species delimitation by bPTP‐ML was consistent with that of jMOTU. bPTP‐BS segregated more groups than bPTP‐ML, which was excluded to avoid overestimations.

## 4 DISCUSSION

In this study, a total of 2,422 individuals representing 143 species of 110 genera in 17 families were recorded from Xinjiang wild fruit forests, China. We found that the lepidopteran insects in Xinjiang wild fruit forests are mainly composed of Noctuidae, Geometridae, Crambidae, Tortricidae, and Erebidae, with species‐rich diversity. The Noctuidae had the highest diversity which is consistent with previous studies (Guo et al., 2017; Jiang et al., 2019; Zha et al., 2016).

Our results show that the fluctuation of the species composition and the diversity of lepidopteran insects at the sampling sites in this study is evident across 3 years. For example, the species diversity of the Lepidoptera community in Xinjiang wild fruit forests show a trend of slight increase followed by a significant decrease from 2017 to 2019. This results from different ecological environments, for example, climatic conditions, landforms, vegetation types, and soil across the 3 years. In particular, lepidopteran insects are expected to show rapid responses to climate change because they are indicators with short life cycles and vary in population size over space and time (Bale et al., 2002). Moreover, Lepidoptera (butterflies and moths) are also a highly diverse taxonomic group, including many pollinators and decomposers, and their biotic interactions with plants and other animals are critical for ecological functioning and ecosystem services (Ayres & Lombardero, 2000; Bradford et al., 2002; Kremen et al., 2007). Therefore, climate changes are considerably related to the biodiversity of lepidopteran insects and their ecological features (Wilson & Maclean, 2011). We also found that the species diversity index of lepidopteran insects in wild fruit forests in 2018 increased compared to the other 2 years, which is likely related to the increase in precipitation during that year resulting in more species diversity and an abundance of host plants that increased in the area providing sufficient food sources for lepidopteran insects. However, due to the lack of data on plant diversity changes in the sample areas during the survey, further investigations in combination with an analysis of climate and other factors are needed in the future.

Additionally, exogenous disturbances (e.g., tourism and animal husbandry) could be an important factor affecting change in community composition and the diversity index in 2019. It is speculated that the area of wild fruit forest decreased and germplasm resources continued to disappear mainly due to ice and snow damage, plant diseases, insect pests, overgrazing, and frequent human activities in the mountainous areas (Cao et al., 2016; Cui et al., 2018; Fang et al., 2018, 2019), resulting in the decline in plant community diversity in wild fruit forests. This in turn leads to the decline in local insect community diversity. In recent studies, the influence of extreme climate and geological disasters such as landslides that occurred frequently in the wild fruit forest and surrounding areas pose a serious threat to the ecological environment of the region (Fan et al., 2020; Shan et al., 2021).

The existence of DNA barcode gaps between species is a strong guarantee for successful DNA barcoding for species identification (Čandek & Kuntner, 2015; Zhao et al., 2014). Ideally, DNA barcodes should be characterized by short fragments, large interspecific variation, and high identification efficiency (Kress et al., 2005; Ren & Chen, 2010; Taberlet et al., 2007). Our analysis using molecular identification based on *COI* barcodes indicates that the mean genetic distance between species is 10 times greater than the mean genetic distance within species, which is consistent with the “10 times rule” of DNA barcoding (Hebert, Ratnasingham, et al., 2003). The existence of significant interspecific and intraspecific barcoding gaps proves that most species can be distinguished using these generated sequences in this study. Similarity, our NJ tree showed that the majority of the 67 barcoded species formed distinctive clusters, confirming the utility of this DNA barcoding method in lepidopteran insect surveillance in the wild fruit forests in Xinjiang. As a rapid and accurate species identification method, DNA barcoding has become more efficient when additional species and populations are included in biodiversity surveys (Sonet et al., 2019). However, small sample sizes and low level of species coverage for barcoding indicate the DNA barcode library established in this study for the local species identification is far from comprehensive. Therefore, more material and further study are needed in the future.

Identification success rate analysis is often used to evaluate the success rate of DNA barcode identification (Yang et al., 2018). How to classify species and which method should be used are commonplace dilemmas in species identification, although diverse methods were described in previous studies (Gao et al., 2021; Hausdorf & Hennig, 2010; Hu, 2019; Marshall et al., 2006; Monaghan et al., 2009; Pons et al., 2006; Sites & Marshall, 2004; Wiens, 2007). In the present study, the usefulness of DNA barcoding identification was tested using five methods based on ABGD, BINs, jMOTU, GMYC, and bPTP. Our ABGD results imply that the MOTUs of the initial partition is less than that of the recursive partition, which is closer to the classification based on morphology. This result is consistent with the theory that a recursive partition is expected to better handle heterogeneity in the dataset, while an initial partition is usually stable on a wider range of prior values and is usually closer to the morpho‐based species identifications (Puillandre et al., 2012). According to Fujisawa and Barraclough (2013), the GMYC single‐threshold model is more reliable than the multiple thresholds model which could lead to an overestimation of the number of OTUs. However, we found that a considerably smaller number of MOTUs was produced in sGMYC than mGMYC, which is most likely due to the small sample size in our dataset (Luo et al., 2018). In addition, 97 OTUs were generated by the BIN system, suggesting that the BIN system tends to overestimate the number of species designated for our dataset, which is consistent with many previous studies (Song et al., 2018; Yang, Landry, et al., 2016; Zhou et al., 2019). In contrast to BINs, both jMOTU and bPTP yielded 71 MOTUs, which are very similar to ABGD designations. In general, our findings indicate that the distance‐based methods (ABGD and jMOTU) and the character‐based method (bPTP) outperform GMYC. BINs is inclined to overestimate species diversity compared to other methods.

Although the lepidopteran DNA barcode database established in this study not only can provide scientific data for the prevention and control of agricultural and forestry pests in the Xinjiang wild fruit forests but it also has an important significance for monitoring changes in local species in the future. However, future applications of this approach should involve barcoding more species and adding other genetic markers that will increase the discriminatory power of this identification method. DNA barcoding could also be utilized with next‐generation sequencing (NGS) to identify large numbers of species at one time (i.e., bulk samples), thereby significantly lowering the processing time involved in species identification (McCormack et al., 2013). Yet, several factors can affect the accuracy of species identification based on mitochondrial *COI* DNA barcodes and metabarcoding, for example, Numts, mitochondrial heteroplasmy, or phylogeography, and should be used with caution in biodiversity surveillance for some groups (Li et al., 2021).

## 5 CONCLUSION

In this study, 2,422 specimens belonging to 143 species of 110 genera representing 17 families were counted and recorded in Xinjiang wild fruit forests. The most abundant family was Noctuidae (54 species), followed by Geometridae (23 species) and Crambidae (13 species). The Shannon diversity index was also highest for Noctuidae in 2018 (2.5198). The majority of the 67 barcoded species formed distinctive clusters, confirming the utility of the DNA barcoding method for insect biodiversity surveillance. We establish a preliminary DNA barcode library for the local species, which is clearly not yet complete and needs to be pursued for assembling a comprehensive barcode reference library. This would then serve in monitoring insect community dynamics by using DNA barcoding as an additional tool for accurate and quick species identification. In this study, five methods, ABGD, BINs, jMOTU, GMYC, and bPTP, were used to classify species. The results of distance‐based methods (ABGD and jMOTU) and the character‐based method (bPTP) outperformed GMYC. BINs overestimated the species diversity compared to other methods. Our results reveal that the diversity of lepidopteran insects is highly susceptible to ecological impacts in the Xinjiang wild fruit forest ecosystem.

## CONFLICT OF INTEREST

All authors have no conflicting interests.

## AUTHOR CONTRIBUTIONS

**Jinyu Zhan:** Writing – original draft (lead); Writing – review & editing (lead). **Yufeng Zheng:** Data curation (equal); Software (equal). **Qing Xia:** Data curation (equal); Software (equal). **Jin Wang:** Investigation (equal); Resources (equal). **Sibo Liu:** Investigation (equal); Resources (equal). **Zhaofu Yang:** Project administration (lead).

## Supporting information

Appendix S1

Appendix S2

Appendix S3

Appendix S4

Supplementary Material

## Data Availability

The data that support the findings of this study are openly available in GenBank of NCBI at: https://www.ncbi.nlm.nih.gov, DNA sequences (GenBank accessions MZ686723–MZ686918). Appendices in the study are available in Dryad (https://doi.org/10.5061/dryad.hmgqnk9jv).
